# Parametric excitation and mode control using an Oersted field in a NiFe nanowire

**DOI:** 10.1038/s41598-021-92149-4

**Published:** 2021-07-09

**Authors:** S. Hwang, Seungha Yoon, Dongpyo Seo, S. H. Han, B. K. Cho

**Affiliations:** 1 School of Materials Science and Engineering, Gwangju Institute of Science and Technology (GIST), Gwangju, 61005 Republic of Korea; 2 Smart Energy & Nano Photonics Group, Korea Institute of Industrial Technology, Gwangju, 61012 Republic of Korea; 3 Division of Navigation Science, Mokpo National Maritime University, Mokpo, 58628 Republic of Korea

**Keywords:** Magnetic properties and materials, Nanowires, Spintronics, Magnetic properties and materials, Magnetic devices

## Abstract

Parametric pumping is a nonlinear wave phenomenon and a promising technique for electronic devices based on spin waves, so-called “magnonics”. For parametric excitation, a magnetic nanowire system that has a built-in dc current line to produce an Oersted field is designed, and for spin wave detection, a micro-Brillouin light scattering (μ-BLS) system is used. A spin wave with a frequency of *f*_sw_ = 5.6 GHz is observed when a pumping microwave with a frequency of *f*_mw_ = 11.2 GHz is applied. The wave is found to be of the *n* = 1 width mode (*n* is the antinode number), and its mode changes to an edge-localized (or possibly *n* > 1) mode when the Oersted field (or current) varies. Joule heating effects are not observed in the pumping process. Thus, spin wave mode control by the built-in current would be a convenient and useful method to enhance the efficiency and compatibility in applications of spin-based electronics.

## Introduction

Parallel parametric pumping is an attractive method for the generation and amplification of spin waves in electronic applications utilizing spin waves as information carriers, so-called magnonics^1–4^. In conventional direct excitation using a microwave field, the width of the antenna and uniformity of the microwave magnetic field limit the characteristics of the excited spin wave^5^. Under this structural restriction, only a spatially symmetric spin wave with a long wavelength can be excited, and the excited wave frequency should be the same as that of the microwave source. On the other hand, parallel parametric pumping excites a spin wave with half the frequency of the microwave field, and the spin wave can have a wide range of wave vectors, including the exchange spin-wave mode with a short wavelength^6,7^. In addition, excitation of antisymmetric waves is possible in parametric pumping, while it is not possible in direct pumping^8^. Parametric pumping is achieved only when the microwave power exceeds a certain threshold value determined by the damping parameter and the ellipticity, i.e., the ratio of the in-plane magnetization components participating in the precession of the system^9–11^. A lower damping parameter and a higher ellipticity result in a higher parametric pumping efficiency.

To date, yttrium ion garnet (YIG)^6,12–17^ and NiFe^8,18–20^ have been widely used for magnonics applications based on the parametric process. In YIG, a spin wave with propagation length of the millimeter scale can be generated because of its low damping parameter, but it has drawbacks in its compatibility with nanosized semiconductor electronics. On the other hand, because the damping parameter of NiFe is larger than that of YIG, the spin wave of NiFe has a propagation length on the micrometer scale. Thus, NiFe is suitable for investigating the dynamic properties of spin waves in nanomagnets. Although the large damping parameter causes a high threshold pumping power, thus lowering the parametric pumping efficiency, this problem can be resolved by reducing the magnetic element size^21^. Indeed, various NiFe micro- and nanostructures have been studied to improve the magnonic application capability. In particular, the wire-type structure is one of the core structures in magnonics for the generation and propagation of spin waves. Parametric pumping is found to be efficient in exciting spin waves in microscopic wire systems^22–24^, and the amplification of the spin wave enhances the propagation length^25,26^. In addition, mode control and tuning of the wave vector obtained by magnetic fields are important in technical applications of parametric spin-wave pumping in wire structures^8,20,22^.

In such confined micro- and nanowire systems, the excited spin wave is quantized along the width, which can be differentiated by the antinode number (*n*)^27^. In particular, the Damon-Eshbach (DE) spin wave of the width-quantized mode propagates along the wire direction with a large group velocity^28^. Thus, excitation of DE spin waves is important in magnetic wire systems and their application. It is also known that the applied field is a useful parameter for controlling the spin-wave property, such as changing the mode^29^. Thus, it is of great interest to study the characteristics of spin waves in nanoscale magnetic wires excited by parametric pumping combined with built-in field application.

Here, we investigate the characteristics of the spin wave induced by parametric excitation in a 650 nm-wide NiFe nanowire, where the magnetic field is adjusted by a built-in dc current line. Spin wave excitation is observed using a micro-Brillouin light scattering (μ-BLS) system. The field dependence of the μ-BLS mode profile shows clear evidence of the wave mode transition from a width-quantized center mode to a localized-edge mode. Joule heating is found to have almost no detrimental effect on the pumping process. Thus, utilization of the dc Oersted field induced by a dc current line is found to be effective and useful for controlling the parametric spin waves in magnetic nanowires.

## Experimental method

**Figure 1:**
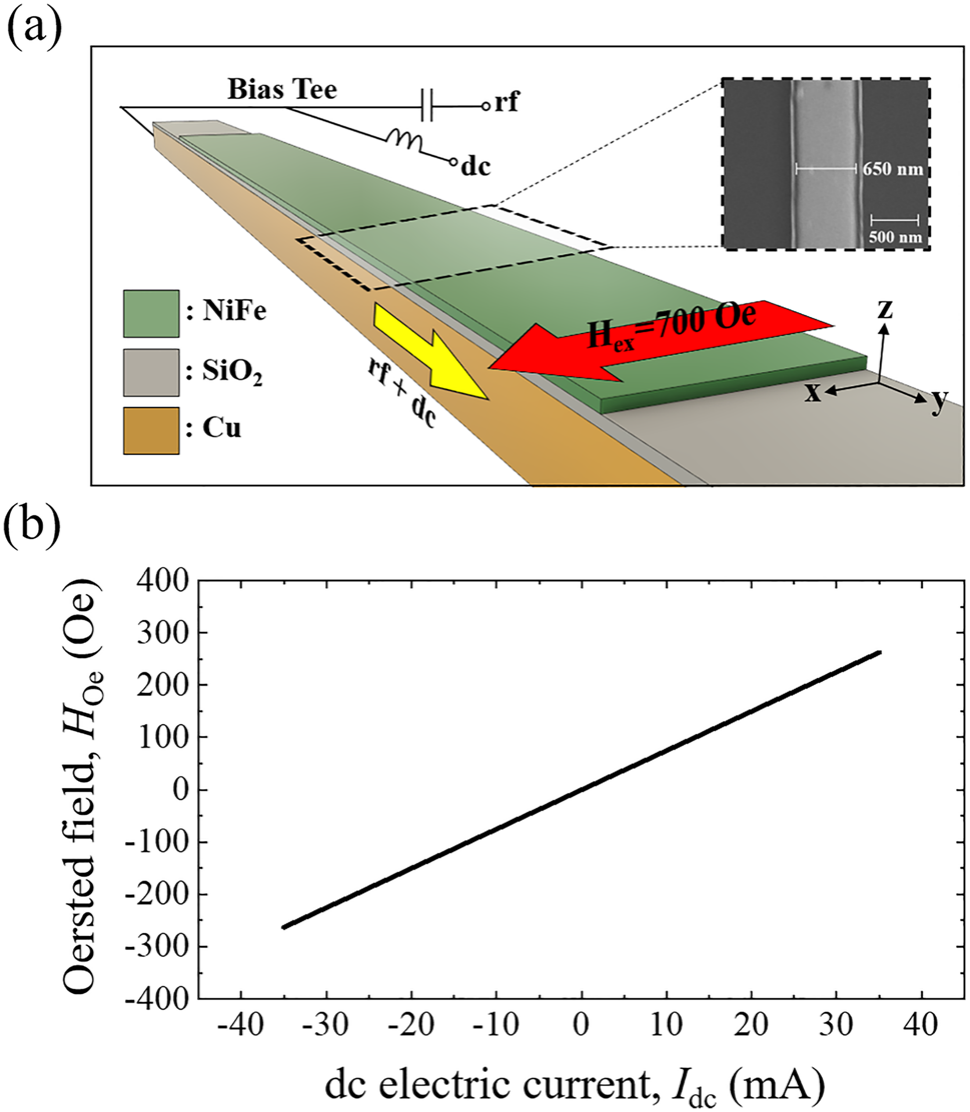
**(a)** Schematic diagram of a permalloy nanowire for parametric pumping. A global external field (*H*_ext_) of 700 Oe is applied along the *x*-axis. The inset presents an SEM image of the top surface in the *x*–*y* plane, showing the topology of the nanowire and the 650 nm width. **(b)** Calculated Oersted field along the *x*-axis as a function of the dc current.

Figure 1a shows a schematic structure of the parametric pumping system employed in this study, and the inset shows a scanning electron microscope (SEM) image of the top surface. The spin wave guide, which consists of Cu(120)/SiO_2_(30)/NiFe(30)/SiO_2_(5), is deposited on an Al_2_O_3_ substrate by a magnetron sputtering system, where the numbers in parentheses are the thickness of each layer in nm. A NiFe nanowire 650 nm in width and 20 μm in length is fabricated by electron-beam lithography and lift-off techniques. The role of the SiO_2_ at the top of the stack is as a capping layer, and that of the SiO_2_ between NiFe and Cu is as insulation. To induce transverse magnetization of the nanowire, the external magnetic field *H*_ex_ = 700 Oe is applied along the *x*-axis, i.e., the width direction. The microwave current, mixed with the dc current by a bias tee, flows in the Cu antenna layer. The pulsed microwave current has a pulse width (${t}_{pulse}$) of 500 ns with an interval time (${\tau }$) of 1 μs to minimize Joule heating. The local Oersted field, *H*_Oe_, produced by the dc current is adjusted to control the magnetization in the NiFe nanowire. As shown in Fig. 1b, *H*_Oe_ is calculated using the formula^30^ $H=\frac{2I}{wd}{\left[U-D\right]\times 10}^{-3}$, where *w* (650 nm) and *d* (120 nm) are the width and thickness of the Cu antenna, respectively. The step functions of *U* and *D* at the NiFe surface (*z* = 60 nm) are calculated to be $U=2\left(z+d\right)\mathrm{arctan}\left[\frac{w/2}{z+d}\right]+\frac{w}{2}\mathrm{ln}\left[{(w/2)}^{2}+{\left(z+d\right)}^{2}\right]$ and $D=2z\mathrm{arctan}\left[\frac{w/2}{z}\right]+\frac{w}{2}\mathrm{ln}[{(w/2)}^{2}+{\left(z\right)}^{2}]$. The total magnetic field, *H*_tot_, is the sum of *H*_ex_ and *H*_Oe_. In our system, the measurement is carried out in the range of 500 Oe ≤ *H*_tot_ ≤ 900 Oe by controlling *H*_Oe_ (equivalently the dc current).

The spectroscopic measurement is carried out using a micro-Brillouin light scattering (*μ*-BLS) system interfacing with a programmable computer^31,32^. The $\mu$-BLS system possesses a microscope objective with a high magnification (100×) and a large numerical aperture (0.75) to obtain a beam spot size of approximately 250 nm in diameter, which significantly enhances the lateral resolution limit. The incident beam, generated by a 532 nm wavelength diode-pumped solid laser (DPSS), is focused on a specific position of the nanowire through the objective lens. The sample is located on an x–y–z piezoelectric stage for deliberate spatial scanning at the nanometer scale to plot the intensity map of the spin-wave mode. The light scattered from the spin wave in the NiFe nanowire passes through a (3 + 3)-pass tandem Fabry–Perot interferometer to yield the frequency domain spectrum. The laser intensity in our experiment is reduced to less than 1 mW to minimize possible thermal effects.

## Results and discussion

**Figure 2:**
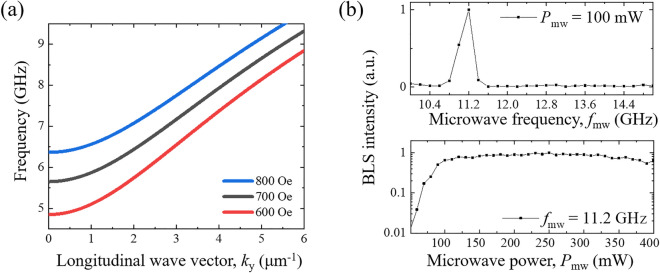
**(a)** Dispersion relation for the spin wave of the n = 1 width mode for fixed magnetic fields of *H* = 600, 700, and 800 Oe. **(b)** Normalized BLS intensity at *H* = 700 Oe as a function of microwave frequency (*f*_mw_) for a fixed microwave pumping power of *P*_mw_ = 100 mW (upper panel) and as a function of *P*_mw_ for a fixed *f*_mw_ = 11.2 GHz (lower panel). The BLS intensity is observed at a frequency of *f*_sw_ = 5.6 GHz, i.e., *f*_sw_ = *f*_sw_/2.

To calculate the dispersion relation of width-quantized modes, the approximate equation is derived from the well-established dispersion equation for an infinite ferromagnetic film^31,33^. The approximation equation expects the inhomogeneous internal field across the width. Considering the width of NiFe layer, a uniform field at the center of NiFe layer is assumed as an internal field. From the analytic process, the dispersion relations of the width-quantized mode with *n* = 1, where *n* is the antinode number of spin waves, are calculated and plotted in Fig. 2a for fixed magnetic fields of 600, 700, and 800 Oe. The dispersion curve shifts up as the field increases.

To identify a parametric process based on the dispersion curves, we measure parallel parametric pumping with *H* = 700 Oe using μ-BLS. The BLS intensity, which is detected at the center of the top surface of the nanowire, is plotted as a function of the excitation microwave frequency (*f*_mw_) for a fixed microwave power of 100 mW (upper panel of Fig. 2b). A peak in the intensity is observed at the frequency of *f*_mw_ = 11.2 GHz, indicating that spin excitation occurs. The measured spin wave frequency (*f*_sw_) is 5.6 GHz, half the excitation frequency, i.e., *f*_sw_ = *f*_mw_/2, which means that the spin excitation is induced by parametric pumping. In addition, the observed spin wave frequency is the same as that at *k*_y_ ≈ 0 in the dispersion curve with *H* = 700 Oe in Fig. 2a. Because the ellipticity decreases as the wave vector *k* increases and thus microwave coupling with spin waves becomes weak, the parametric pumping should occur at a low *k* value^10,22^. Thus, the data show clear evidence that a width-quantized spin wave of the *n* = 1 mode is successfully excited in the nanowire system in Fig. 1 through parametric pumping. The BLS intensity is also plotted as a function of the excitation microwave power (*P*_mw_) with a fixed microwave frequency of 11.2 GHz (lower panel of Fig. 2b). The applied power is in the range of 50 mW ≤ *P*_mw_ ≤ 400 mW with a 10 mW step. The BLS intensity increases exponentially at ≈ 80 mW, which represents the threshold power for parametric pumping. Because it is evident that parametric pumping with a microwave of *f*_mw_ = 11.2 GHz excites a spin wave in our system, the measurements to study the spin wave properties below are performed at the same *f*_mw_ = 11.2 GHz, and the BLS signals are obtained at *f*_sw_ = 5.6 GHz.

**Figure 3:**
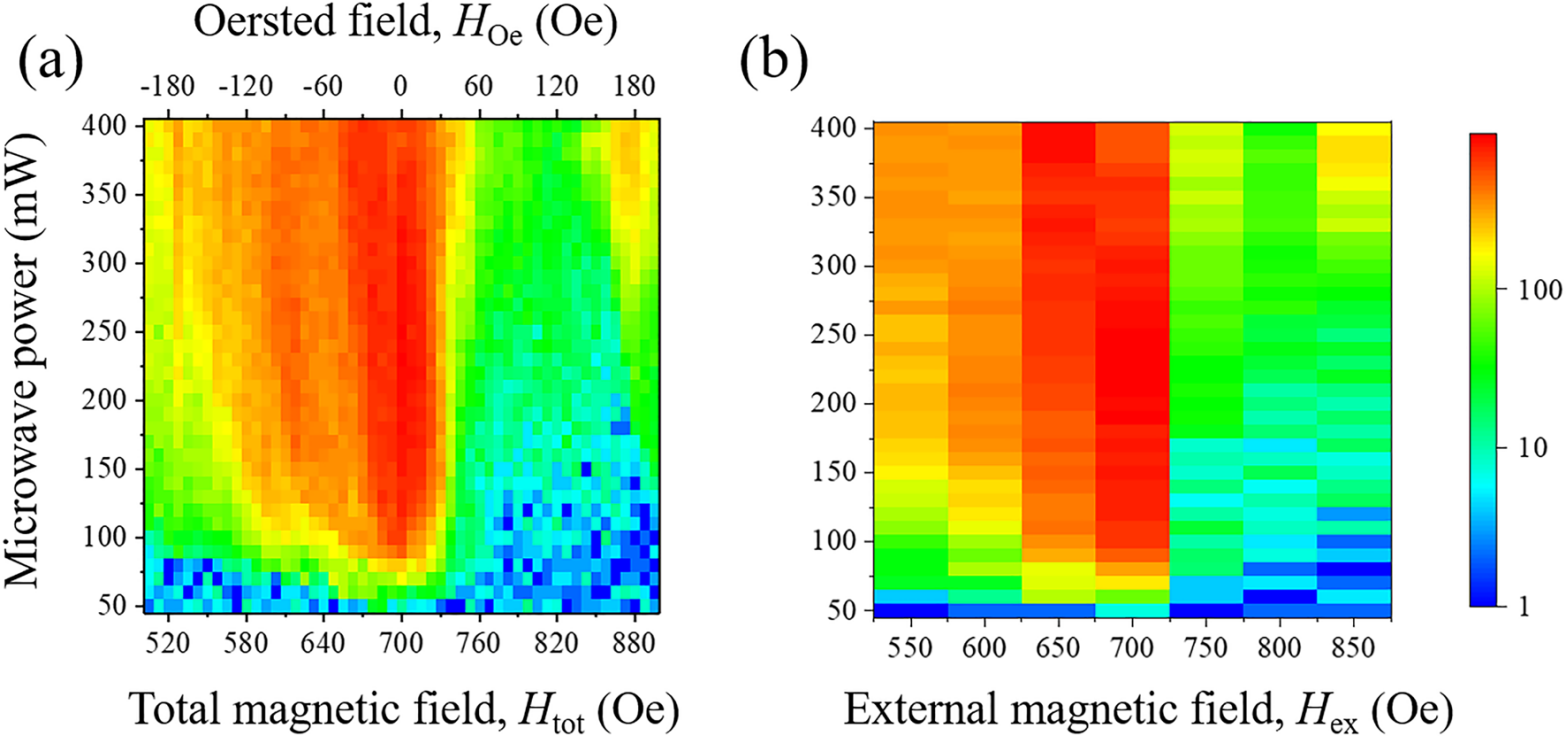
Threshold characteristics of parallel parametric pumping, i.e., BLS intensity as a function of the microwave power with a fixed frequency (*f*_mw_ = 11.2 GHz) and the magnetic field: **(a)** the Oersted field and total field are controlled by the dc electric current; **(b)** an external static field is applied in a range of 550 Oe ≤ *H* ≤ 850 Oe with a 50 Oe step. The color scale represents the BLS intensity of the spin wave with a frequency of *f*_sw_ = 5.6 GHz.

To investigate the characteristics of the threshold pumping power in terms of the applied field, the dc current is swept from -26 to 26 mA with a 1 mA step together with the RF current at a fixed frequency. Figure 3a shows the threshold characteristics as a function of the field, where the threshold values are observed to follow an asymmetric butterfly curve^1,22^. This means that the field dependence varies depending on whether the threshold value is on the higher- or lower-field side of the total magnetic field (or current) of the lowest threshold value. For example, the threshold value is lowest at *H*_tot_ = 700 Oe and rapidly increases as the total field increases, while the threshold increases at a substantially lower rate as the field decreases. For comparison, Fig. 3b shows similar data when the field variation is induced only by external field application in a range of 550 Oe ≤ *H* ≤ 850 Oe with a 50 Oe step. Even though a one-to-one comparison is not possible, the gross features in both cases are observed to be identical. This shows that the effect of Joule heating by a current on the parametric process is affordable in that utilizing an Oersted field created by a dc electric current can replace utilizing an external magnetic field. The additional simulation data on heating effect can be found in the Supplemental Material.

**Figure 4:**
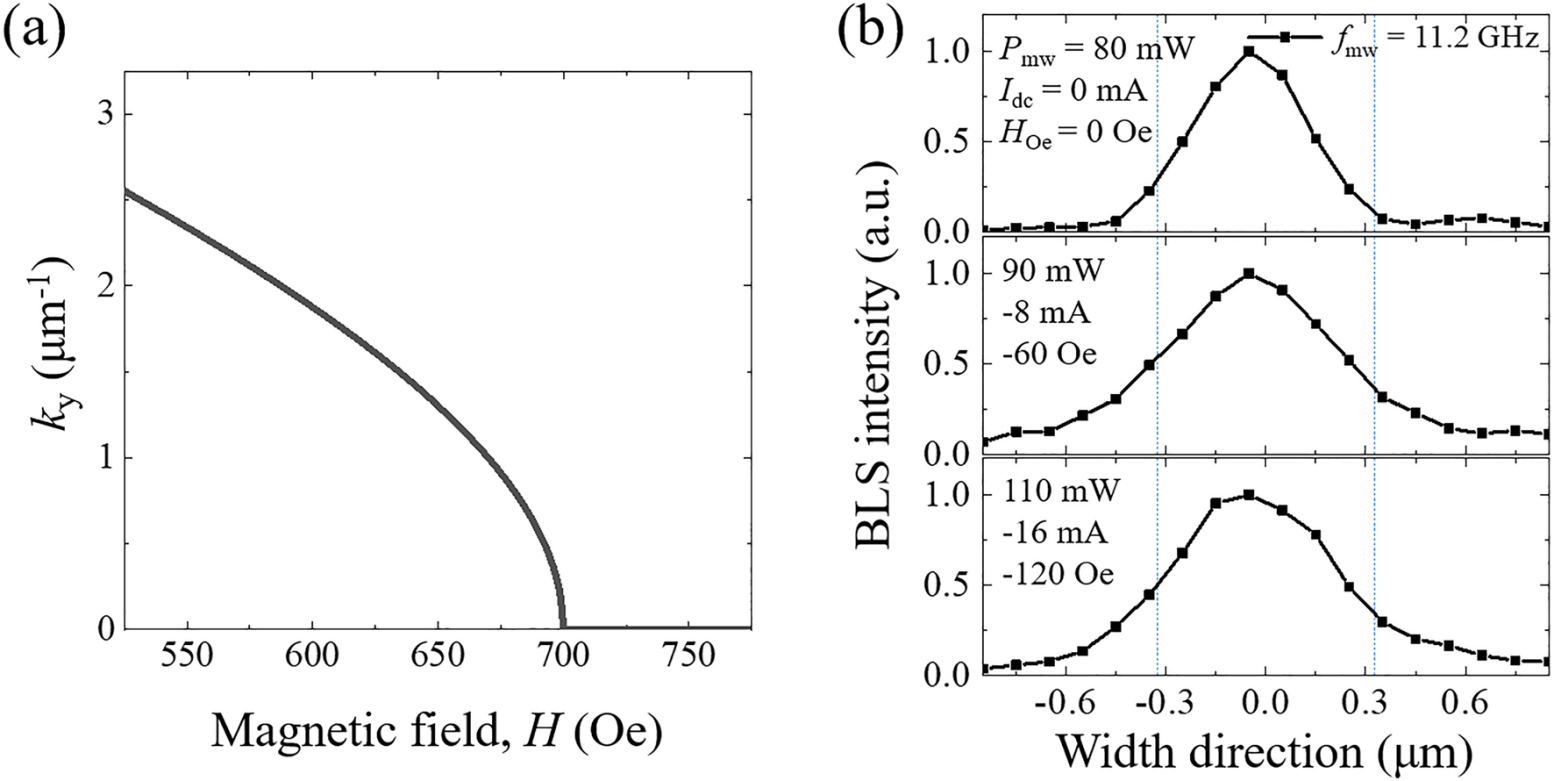
**(a)** Field dependence of the longitudinal wave vector *k*_y_ at a fixed frequency of *f*_sw_ = 5.6 GHz. **(b)** Mode profiles along the width direction. BLS intensities are measured at the frequency of *f*_sw_ = 5.6 GHz. The measurement conditions of the microwave power (*P*_mw_), dc current (*I*_dc_) and Oersted field (*H*_Oe_) are shown in each profile. The microwave frequency (*f*_mw_) is 11.2 GHz.

Figure 4a shows the field dependence of the longitudinal wave vector *k*_y_ at a fixed frequency of *f*_sw_ = 5.6 GHz. For the *n* = 1 width mode, *k*_y_ is zero at *H*_ex_ = 700 Oe and increases as the field decreases. This indicates that the spin wave of the *n* = 1 width mode with *f*_sw_ = 5.6 GHz can be excited in the field region below *H* = 700 Oe, while this excitation is prohibited in the field region above *H* = 700 Oe. In the high field region, the spin wave with *f*_sw_ = 5.6 GHz is expected to be of a higher-order width mode (*n* > 1) if there is one or of a localized-edge mode.

To verify the above scenario, the transverse mode profile is measured along the width direction of the nanowire. Each profile is obtained from spots of $(x\times y)=(20\times 20)$ by spatially resolved μ-BLS with 100 nm and 200 nm step sizes along the width and length directions, respectively. For precise observation of the mode transition, which depends on the field, the pumping condition of each profile is near the threshold power *P*_th_. This is because a pumping energy above *P*_th_ would induce competition between various dipole-dominant modes and, eventually, flow into the fundamental mode with the lowest value of *k*_*y*_^8^. Figure 4b shows the mode profile with a total field *H*_tot_(= 700 + *H*_Oe_) ≤ 700 Oe along the width direction under the measurement conditions of *P*_mw_, *I*_dc_ and *H*_Oe_ specified in the figures. All three profiles clearly show the features of the *n* = 1 width mode spin wave, which is consistent with the expectation. In addition, the threshold power for spin excitation is found to increase as the total field decreases. It is well known that a large wave vector induces a reduction in the mode ellipticity and thus an increase in the pumping power. Because the *k* value increases as the field decreases (Fig. 4a), the pumping power change for the excitation is also consistent with the scenario of the spin wave with the *n* = 1 width mode.

**Figure 5:**
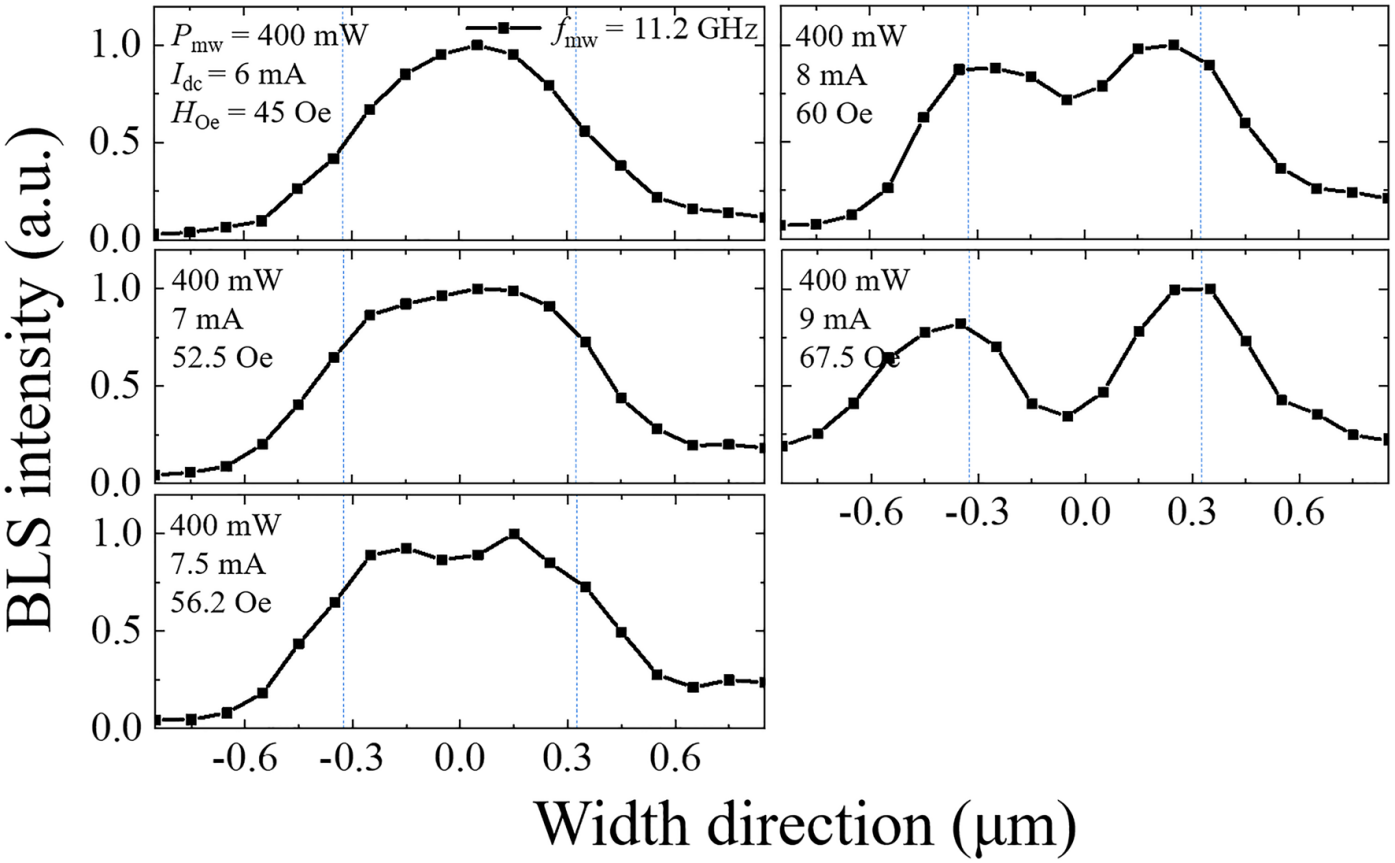
Mode profiles along the width direction. BLS intensities are measured at a frequency of *f*_sw_ = 5.6 GHz. The measurement conditions of the microwave power (*P*_mw_), dc current (*I*_dc_) and Oersted field (*H*_Oe_) are shown in each profile. The microwave frequency (*f*_mw_) is 11.2 GHz.

A similar mode profile measurement is performed in a high field region of *H*_tot_ > 700 Oe and plotted in Fig. 5. Under the conditions of *I*_dc_ = 6 and 7 mA (equivalently, *H*_oe_ = 45 and 52.5 Oe), the mode profile noticeably spreads out, while its shape is similar to that in Fig. 4b. For *I*_dc_ = 7.5 mA, two antinodes (*n* = 2) begin to appear along the width direction, and their shape develops with a further increase in the dc current. Actually, the mode profile with *I*_dc_ = 9 mA reveals the clear characteristics of a localized-edge mode. It is known that the frequency near *k*_y_ ≈ 0 decreases as the antinode number increases until the exchange energy interaction between neighboring spins is large enough to overcome the dipolar energy^5,22^. For the edge mode, the entire dispersion curve is lower than that for the width modes because the internal field is drastically reduced by the demagnetization effect^34,35^. Thus, the microwave power of *P*_mw_ = 400 mW, much larger than those in Figs. 2 and 3, is necessary to observe a noticeable intensity of the BLS signal. The observed mode profile and dispersion calculation strongly support that the built-in dc current can be utilized as an effective and useful tool for the manipulation of the spin wave, such as mode transition, excited by parametric pumping.

## Summary

Parametric pumping is utilized to investigate quantized spin excitation in a wire-type magnetic element of 650 nm in width. Spin waves are found to be excited with a microwave with a frequency of *f*_mw_ = 11.2 GHz and are detected based on the μ-BLS spectrum with a frequency of *f*_sw_ = 5.6 GHz. The field dependence of the threshold power shows butterfly-shaped curves, i.e., an asymmetric field dependence of the threshold power: a gradual increase in threshold power in the low field region and a rapid increase in the high field region. The mode profile along the width direction, together with the analytic dispersion relation, indicates that the spin wave in the low field region is of the *n* = 1 width mode. On the other hand, an edge-localized mode (possibly a high-order width mode, *n* > 1) is observed in the high field region. Thus, it is possible to induce a mode transition of parametric spin waves using the Oersted field created by a built-in current line. This means that the Oersted field (equivalently current) can act as a switch for parametric spin excitation at fixed pumping power and frequency. In addition, because the spin wave has a strong mode dependence of its physical properties, such as transmission, processing speed, and microwave coupling, mode selectivity by an Oersted field would be a great addition in the application of parametric spin waves.

## Supplementary Information

Supplementary Information.
