# Supplementary material for: Parametric excitation and mode control using an Oersted field in a NiFe nanowire
Source: Sci Rep. 2021 Jul 9;11:14207. doi: 10.1038/s41598-021-92149-4 (PMC8270930; doi:10.1038/s41598-021-92149-4)
Supplement: Supplementary file 1 — Supplementary Information. [file 41598_2021_92149_MOESM1_ESM.pdf]

Supplementary Material

Parametric excitation and mode control using an Oersted field in a NiFe nanowire

S. Hwang[a,b], Seungha Yoon[b], Dongpyo Seo[a], S. H. Han[c], and B. K. Cho*[a]

[a]School of Materials Science and Engineering, Gwangju Institute of Science and Technology (GIST), Gwangju 61005, Republic of Korea

[b]Smart Energy & Nano Photonics Group, Korea Institute of Industrial Technology, Gwangju 61012, Republic of Korea

[c]Division of Navigation Science, Mokpo National Maritime University, Mokpo 58628, Republic of Korea

*Email: chobk@gist.ac.kr, Tel.: +82-62-715- 2318, Fax: +82-62-715-2304

On the Joule heating effects due to dc current

In order to have the information on Joule heating due to the dc current for Oested field creation, a simulation was performed using “COMSOL multiphysics”. The same geometrical parameters as in Fig. 1 and a resistance of 6.5 ohms for Cu wire were used for the simulation. It is found that the temperature at the system for parametric excitation is estimated to be 38.5, 41.6, and 46.1 °C when the current of 15, 25, and 35 mA flows in Cu wire, respectively, as shown in Fig. S-1. The temperatures is quite low, compared with the Curie temperature (≈ 546 °C) of NiFe alloy so that the possible Joule heating effect can be ignored in the analysis of the BLS data.

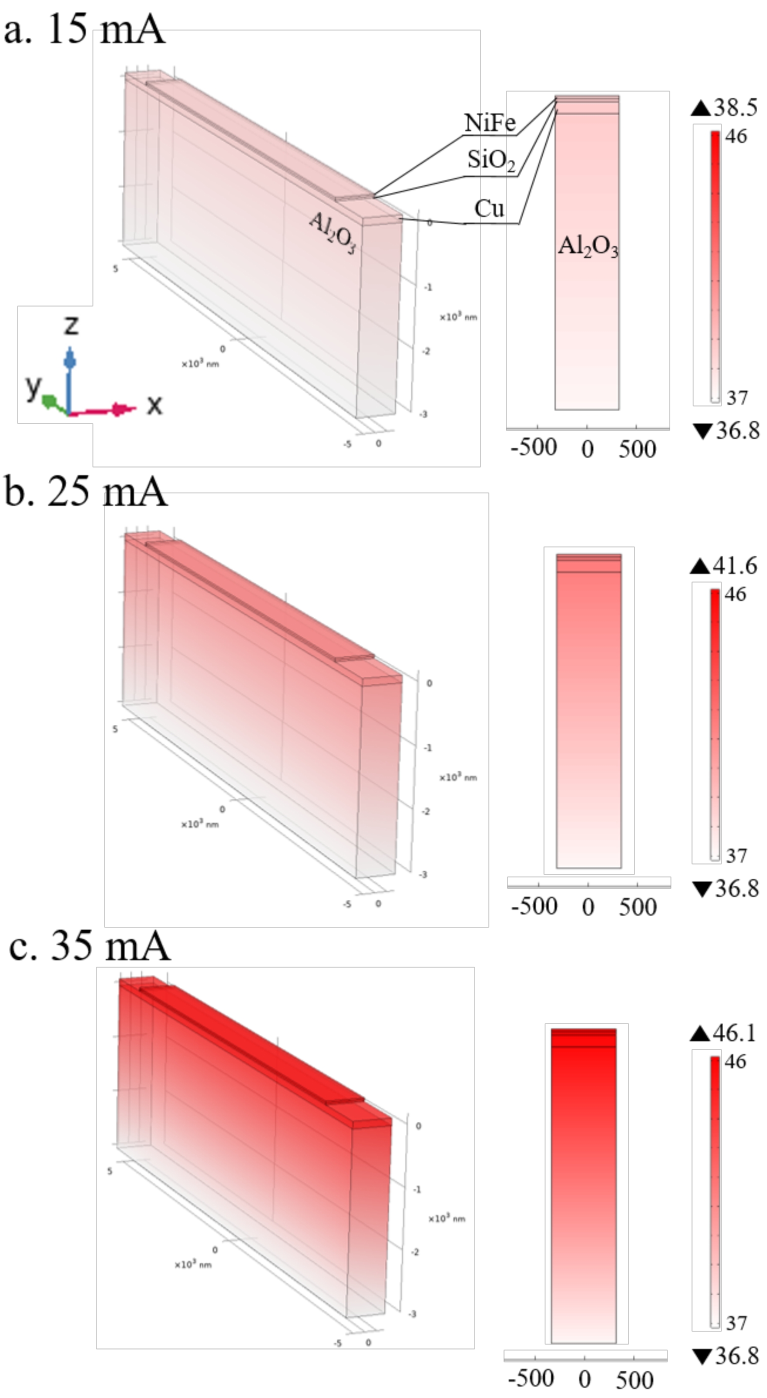

Figure S-1. The temperature gradient and actual temperature of the experimental sample, depending on the magnitude of I_{dc} of: a. 15 mA, b. 25 mA, and c. 35 mA. Simulation is performed using “COMSOL multiphysics”.
